# Is fighting against COVID-19 enough?

**DOI:** 10.1177/1403494820969539

**Published:** 2020-11-12

**Authors:** Walter Kofler, Oleg S. Glazachev, Heidi Lyshol, Gunnar Tellnes

**Affiliations:** 1 Medical University of Innsbruck, Austria; 2 I.M. Sechenov Moscow State Medical University (Sechenov University), Russia; 3 Department of Health and Inequality, Norwegian Institute of Public Health, Norway; 4 Department of Community Medicine and Global Health, University of Oslo, Norway; 5 School of Health Sciences, Kristiania University College, Norway

**Keywords:** COVID-19, public health, infectious diseases, community medicine, health promotion and prevention, NaCuHeal, Nature-Culture-Health

## Abstract

Tools of empirical epidemiology have been and are indispensable to focus political power on blocking the spreading of coronavirus disease 2019 (COVID-19) by stopping transmission. The present paper is a comment on E. Gibney’s article ‘Whose coronavirus strategy worked best?’ (*Nature* 2020;581:15–6). The strategy for phase 2 should be more complex and interdisciplinary than described in the paper in *Nature*, especially in the period before a vaccine and specific treatments are available. The focus on reducing the mortality of COVID-19 will have side effects, including excess mortality from other causes. A part of this excess mortality will be based on the reduction of health-care offers as a consequence of the pandemic, and on structural limitations of the health-care system. A special challenge is to understand the relationship between death *from* and death *with* COVID-19, and therefore the relevance of severe acute respiratory syndrome coronavirus 2 infection in people with pre-existing burdens, for example coronary heart disease, cancer or older age. There is a need to extend the recently used tools to all available instruments, including physiological principles of prevention and promotion. The way to integrate global solidarity into the strategies of the different countries is critical not only for global health but also for the peace and long-term success for each individual country. The consequences of efforts against COVID-19 and the impact on reduced air pollution and climate change are also important to analyse from a global health perspective.

## Introduction

The current situation with coronavirus disease 2019 (COVID-19) is restricted by relevant but insufficient tools. This was demonstrated in a paradigmatic way by the paper ‘Whose coronavirus strategy worked best? Scientists hunt most effective policies’. This excellent paper gives us information about forward-looking international studies on the basis of experimental epidemiology to ‘find ways to identify the measures that best predict infection rates’ and to ‘be able to forecast how adding and removing interventions would change the number of infections on SARS-CoV-2 over time’ [1]. We need such tools. However, the reader can get the impression that it is the responsibility of the scientists to ‘hunt the most effective policies’ and that the complexity of the situation can be handled just with the tools of experimental epidemiology and can be summarised by the position: ‘Without vaccine or effective treatment, stopping transmission remains the only defence against COVID-19’.

Is such a strategy really sufficient, even in phase 2 – a situation in which the reproduction (R) rate is permanently <1, the number of new infected persons is much lower than the number of cured persons, contact tracing is sufficient and there are plenty of empty intensive care unit (ICU) beds for COVID-19 patients? In addition, is this sufficient to handle this epidemic that is also a pandemic? Are we adequately prepared to deal with the next pandemic – hopefully without a lockdown?

## The responsibility of politicians, medical experts and other personnel

The relationship between political decision makers and medical experts is clear: the politicians have to fix the strategy, usually based on unclear prerequisites and unknown future consequences. Nevertheless, they have to decide *now*. They are also responsible for the measures taken. The responsibility of the experts in infectious diseases and public health is to give information based on scientific principles. The scientific information may be expressed mathematically.

The position of the medical doctors (MDs), such as general practitioners, public-health physicians, epidemiologists and specialists in infectious disease, is more complex. Health depends on many factors, which may belong to many other scientific disciplines than medicine – from physics to socio-economics. Up to now, these disciplines have not been compatible on a causal level. The results are often based on averages. The medical expert has to balance all these aspects with the focus on comprehensive proposals of curative, preventive and health-promoting efforts. The MDs need to cooperate with experts from different scientific disciplines, and they have to integrate multi-causality and multi-intentionality to keep in mind many different needs, demands and risks at the same time. MDs have to adjust the proposals according to the progress of the prior measures. Therefore, aspects which are less relevant in phase 1 of an epidemic may gain relevance in phase 2.

### Unintended side effects should be monitored: the example of excess mortality

Excess mortality has not been sufficiently studied. Banerjee et al. recently published an alarming population based cohort study on excess 1-year mortality associated with the COVID-19 pandemic [2] using data from the Office for National Statistics of England and Wales [3]. The Office for National Statistics reported 6000 excess deaths registered in the 2 weeks from 28 March to 3 April 2020, of which about 2500 deaths did not have COVID-19 recorded on the death certificates. We know that severe acute respiratory syndrome coronavirus 2 (SARS-CoV-2) has a higher R_0_ and a higher case fatality rate than the 1918–1919 pandemic of Spanish Influenza, which caused about 50 million deaths [4]. The excess mortality of these other 2500 deaths requires our attention: the curves for ‘total mortality’ and ‘influenza and pneumonia’ in weeks 1–12 of 2020 and the 5-year-averages are totally unremarkable – in contrast to the data for weeks 13 and 14. Banerjee et al. add the excess deaths from the COVID-19 pandemic in those *affected* (indirectly, not infected) by reduced access to health services, the physical, psychological and social effects of distancing and economic changes to those *infected* (direct effects) [2].

### The need for better registration of causes of death and distribution of medical resources

It makes sense to distinguish between two groups of ‘affected deaths’. The first group comprises excess deaths without SARS-CoV-2 infection, caused by the reasons listed above. The improved strategy has to include improved distribution of ICU beds, personal protective equipment, hospital personnel and so on. The second group consists of excess deaths with SARS-CoV-2, but with another diagnosis on the death certificates. These cases call for a deeper analysis of the pathophysiological processes.

### The added burden of COVID-19

Health is a process in which the organism can balance the different demands to homeostasis. Death is the consequence when the demands to the organism cannot be adequately balanced. Therefore, survival and, finally, healing are based on two prerequisites: (a) sufficient available energetical, structural and morphological resources and (b) the sufficient organisation of these resources [5].

This lack of balance can cause a feeling of unwellness or pain, but it need not do so: COVID-19 patients with extraordinarily low blood oxygen levels have reported feeling comfortable [6]. We should not overestimate the subjective feeling as adequate stimulus to visit a physician, who could diagnose an aberration. The person could live decades with a known or unknown pathophysiological deviation because of adequate potential to balance it. We know this from arthrosclerosis for example. These pathophysiological processes explain why a co-morbidity with arthrosclerosis is linked with a high risk of dying of COVID-19. The related principle may be used to explain the excess mortality of different types of natural and man-made disasters, after Chernobyl, Bhopal, Seveso, heat waves, earthquakes and so on [7]. This also explains the formerly unexplained deviation of the mortality distribution of the victims of Hiroshima and Nagasaki [8].

## A systematic analysis of the underlying factors

A comprehensive analysis is missing. Useful proposals have been made by Harvey Fineberg [9] and others. Additional examples will be discussed here.

### Modification of the characteristics of SARS-CoV-2: seasonality?

There may be a slight seasonal effect, like in influenza epidemics, which leads us to expect a reduction in infections and the danger of second and third waves later. Nevertheless, our recent knowledge does not give real hope that SARS-CoV-2 will disappear like Middle Eastern Respiratory Syndrome (MERS). Even with the disappearance of SARS-CoV-2, we should take into consideration that there are many possible candidates that could mutate to a similar virus. Any future strategy should use the given window of opportunity for better preparedness in our health-care systems and communities. This is needed independently of the hope for a vaccine or treatment [10].

### Comprehensive understanding of preventive, health-promoting and curative options

We present just a few examples from a wide field of options:

*Interrupting the chain of infection*: Direct contact with the virus is not enough to cause symptoms. The rate of people with positive tests but without symptoms confirms the assumption that processes must take place before symptoms occur. The fact that people without symptoms can infect others is another argument to consider tools to interrupt the process after immediate contact with the virus and its ‘implementation’ into the host body. The ongoing research, for example with N-Chlortaurin as antiseptic, should be followed with keen interest [11,12].
*Health promotion: the example of intermittent hypoxic–hyperoxic training*: The ability to use oxygen in the air and to transport it to the needed organs/tissues decreases in the elderly, and this decrease is also a consequence of common diseases (e.g. coronary heart disease (CHD)). Intermittent hypoxia training or hypoxia–hyperoxia conditioning technologies are tools to improve oxygen uptake with positive effects on, for example, CHD [13] and Alzheimer’s disease [14]. A critical factor for the coping capacity of patients with SARS-CoV-2 is the amount of available oxygen. Hypoxic–hyperoxic training may serve to improve life in people predisposed to respiratory infections, with a high risk of developing chronic non-infectious diseases, as well as for the rehabilitation of patients after COVID-19, but also as a preventive tool.

The Nature-Culture-Health model (NaCuHeal) is another example of health promotion, which has been shown to improve health, quality of life and function [15].

## COVID-19: a pandemic – not just an epidemic

Each country has special conditions and therefore needs its own special strategy. The success of each country also depends on the success of all the other countries which suffer from the pandemic. The way in which this is handled is critical, not just for the global success of finally eliminating SARS-CoV-2. It also influences the freedom to travel and to exchange goods in the globalised economy, and strongly influences local and global health because of the consequences on morbidity and mortality as effects of unemployment and so on.

In the recent situation, we are in danger of focussing on short-term wins, as was common in the 19th and large parts of the 20th century. The use of international agreements and treaties was comparable with the principles of game theory: reciprocation, only as long as the individual win can be maximised! At the end of the 20th century and the beginning of the 21st century, clever politicians have recognised that the long-term win for each country is higher if the agreements can be accepted and controlled independently from the actual and short-term win. It would in the end be more economical to support poor countries, even in the form of gifts: the costs to repair stability and regain predictability would be much higher than the costs to balance given inequalities.

The long-term consequences of efforts against COVID-19 and the positive impact of the ongoing pandemic on reduced air pollution and climate change are also important to analyse from a global health perspective. Global health and the interplay between nature, culture and health (NaCuHeal) may decide the future of the global economy and the fate of human beings on our planet [16]. There is an untapped potential for improving public health by employing health-promoting nature and cultural activities in the local community [17–19]. The goal is an increased ability to cope, productivity and prosperity to *all* people, that is, not only the affluent members of society, but also the ones who are in danger of becoming permanently incapable of working.

The next level of the argument would be to respect the neglected, maybe suppressed conclusions of Darwin: he proposed that the natural progress from the purely biological understanding of other species would be understanding them as moral creatures [20]. The evolutionary process would logically cause the extension of sympathy not just to ‘the men of all nations and races . . . but to the humblest living creature’. Therefore, the way we handle the recent pandemic can give us the tools to improve not only the level of health, but also the guidance for socio-ecological and cultural-based peace and sustainable development [21]. The importance of health promotion and contact with nature are also underlined by the World Health Organization that recently published a set of prescriptions for a healthy and green recovery from COVID-19, of which the first prescription is to ‘protect and preserve the source of human health: Nature’ [22].

## The need for a holistic perspective on pandemics, climate change and global public health

The recent lockdown has been influenced by the lack of preventive activities in consequence of the experiences with SARS and MERS. We have known that the next pandemic would come – sooner or later. We may be able to develop a specific vaccine and treatment against SARS-CoV-2. This would not be sufficient to prevent the next lockdown. We know from history how important general improvements in health are (e.g. the decrease in the mortality rate of tuberculosis even without a vaccine and without a specific drug). Such processes take time. Therefore, we have to start now and not only with tools based on classic physiology and contact tracing. Phase 3 is needed. The necessary structure must be integrated into other strategies to deal sufficiently with the known challenges, for example global warming, climate change, the mass death of bees and so on.

## Conclusion

The present situation without a vaccine and specific treatments should stimulate a systematic focus on saving lives using curative, preventive and health-promoting tools. The available knowledge should be used for additional research for a better understanding of the combined effects between different diseases, but also between the interactions of biological, physical, emotional, cognitive and intellectual challenges.

The way to integrate global solidarity into the strategies of the different countries is critical not only for global health but also for the peace and long-term success of each individual country. The consequences of efforts against COVID-19 and the impact on reduced air pollution and climate change are also important to analyse from a global health perspective. There is an urgent need to extend the activities from phase 1 – coping with the acute pandemic – and phase 2 –to be prepared for a second wave of COVID 19 and to develop a specific vaccine and treatment – to phase 3 – to be prepared for the next pandemic, which must also be balanced with other expected fundamental risks to our existence. Therefore, there is a need to extend the recently used tools to all available instruments, including physiological principles of prevention and promotion.

## References

[1] Gibney E. Whose coronavirus strategy worked best? Scientists hunt most effective policies. Nature 2020;581:15–6. 10.1038/d41586-020-01248-1 32341558

[2] Banerjee A Pasea L Harris S, et al Estimating excess 1-year mortality associated with the COVID-19 pandemic according to underlying conditions and age: a population-based cohort study. Lancet 2020;395:1715–25. 10.1016/S0140-6736(20)30854-0 PMC7217641 32405103

[3] Office of National Statistics, Death registered weekly in England and Wales: provisional: week ending 3 April 2020, https://www.ons.gov.uk/peoplepopulationandcommunity/birthsdeathsandmarriages/deaths/datasets/weeklyprovisionalfiguresondeathsregisteredinenglandandwales (accessed 7 September 2020).

[4] Layne SP Hyman JM Morens DM, et al New coronavirus outbreak: framing questions for pandemic prevention. Sci Transl Med 2020;12:eabb1469. 10.1126/scitranslmed.abb1469 PMC11000442 32161107

[5] Dussault AC Gagné-Julien AM. Health, homeostasis, and the situation-specificity of normality. Theor Med Bioeth 2015;36:61–81. 25604955 10.1007/s11017-015-9320-1

[6] Couzin-Frankel J. The mystery of the pandemic’s ‘happy hypoxia’. Science 2020;368:455–6. 10.1126/science.368.6490.455 32355007

[7] Kofler W. Health effects of environmental disasters and the need of a more complex model of man. In: Steinberger Y (ed) Preservation of our world in the wake of change. Jerusalem: ISEEQS VI A/B, 1996, pp.275–82.

[8] Kofler W Lercher P Puritscher M The need for sufficiently taking into account unspecific effects in the understanding of health risk: Part 1: Unexplained phenomena; Part 2: Epistemological limitations and offers for solution; Part 3: Prove of the proposed solution by an experimentum cruces. IUAPPA and Korean Society for Atmospheric Environment, Seoul (on CD-ROM), 2001. F 0245a-c.

[9] Fineberg H. Ten weeks to crush the curve. New Engl J Med 2020;382:e37. 32237671 10.1056/NEJMe2007263

[10] Neher RA Dyrdak R Druelle V, et al Potential impact of seasonal forcing on a SARS-CoV-2 pandemic. Swiss Med Wkly 2020;150:112. 10.4414/smw.2020.20224 32176808

[11] Gottardi W Nagl M. N-chlorotaurine, a natural antiseptic with outstanding tolerability. J Antimicrob Chemother 2010;65:3. 10.1093/jac/dkp466 20053689

[12] Arnitz R Stein M Bauer P, et al Tolerability of inhaled N-chlorotaurine in humans: a double-blind randomized phase I clinical study. Ther Adv Respir Dis 2018;12:1753466618778955. 10.1177/1753466618778955 PMC5985600 29857780

[13] Glazachev O Kopylov P Susta D, et al Adaptations following an intermittent hypoxia-hyperoxia training in coronary artery disease patients: a controlled study. Clin Cardiol 2017;40:370–6. 10.1002/clc.22670 PMC6490434 28323322

[14] Bayer U Likar R Pinter G, et al Intermittent hypoxic–hyperoxic training on cognitive performance in geriatric patients Alzheimer’s and dementia. Alzheimers Dement (N Y) 2017;3:114–22. 10.1016/j.trci.2017.01.002 PMC5651371 29067323

[15] Batt-Rawden KB Tellnes G. Nature–Culture–Health activities as a method of rehabilitation: an evaluation of participants’ health, quality of life and function. Int J Rehabil Res 2005;28:175–80. 10.1097/00004356-200506000-00013 15900190

[16] Tellnes G. How can nature and culture promote health? Scand J Public Health 2009;37:559–61. 10.1177/1403494809342373 19666672

[17] Tellnes G. President’s column: health promotion in the local community. Eur J Public Health 2005;15:331–3.

[18] Tellnes G Batt-Rawden KB Christie WH. Nature-Culture-Health promotion as community building. Herald IAS.RS 2018;1:15–20.

[19] Tellnes G Batt-Rawden KB Christie WH. Public mental health promotion, epigenetics and NaCuHeal – activities. Herald IAS.RS 2019;1:66–73.

[20] Darwin C. The descent of man, and selection in relation to sex. 2nd ed., 12th thousand, revised and augmented. London: John Murray, 1877.

[21] Kofler W Glazachev O Tellnes G. Evolution and the promotion of health, wellbeing and peace (instead of preface). Herald IAS.RS 2020;1:1-1.

[22] World Health Organization. Nature is our greatest source of health and well-being. WHO celebrates World Environment Day 2020, for nature and for people, https://www.who.int/news-room/feature-stories/detail/world-environment-day-2020 (accessed 7 September 2020).

